# Effect of Platinum Content on Properties of CNT-Supported Pt–Mo Catalyst for Ethanol Electrooxidation Reaction

**DOI:** 10.3390/nano16090552

**Published:** 2026-04-30

**Authors:** Oleg Korchagin, Marina Radina, Alexey Kuzov, Vladimir Andreev, Andzhela Bulanova

**Affiliations:** 1 Frumkin Institute of Physical Chemistry and Electrochemistry, Russian Academy of Sciences, Moscow 119071, Russia; merenkovamarina@mail.ru (M.R.); scourge@mail.ru (A.K.); vandr@phyche.ac.ru (V.A.); 2 Department of Physical Chemistry and Chromatography, Samara University, Samara 443086, Russia; av.bul@yandex.ru

**Keywords:** nanodispersed electrocatalysts, carbon nanotubes, ethanol electrooxidation, alcohol fuel cell

## Abstract

The CNT-supported nanodispersed Pt–Mo catalysts for the ethanol electrooxidation reaction in the alkaline solution are synthesized and their characteristics are studied. Based on the XPS studies in a wide range of platinum content (10–40 wt %), it is found that in the composition of the catalysts, platinum is predominantly in the metallic state, and molybdenum is in the hexavalent form, probably in the form of MoO_3_ oxide. According to the XRD and electrochemical studies, the Pt/CNT and PtMo/CNT catalysts with equal platinum contents (~20 wt %) are characterized by similar platinum crystallite sizes (5–10 nm) and electrochemically accessible surface areas (23–26 m^2^/g_Pt_). This indicates that platinum is not shielded by the molybdenum compounds. When the platinum content increases above 20 wt %, the Pt:Mo atomic ratio increases (the nominal ratio is 1:1), which may be due to the decoration of molybdenum oxide with platinum nanoparticles. A study of the kinetics of the ethanol electrooxidation reaction showed that the activity of the PtMo/CNT system is higher than that of the Pt/CNT catalyst. However, the efficiency of platinum use decreases as its content in the PtMo/CNT system increases from 10 to 40 wt %. On the other hand, the systems containing 20–40 wt % Pt exhibit the highest activity per unit catalyst weight, making them very promising for use as a component of the anode active layer of a fuel cell. The tests of the alkaline ethanol fuel cell based on the synthesized catalysts show the maximum power density of 29 mW/cm^2^, which corresponds to the level of the best literature parameters under similar experimental conditions.

## 1. Introduction

**Figure 1:**
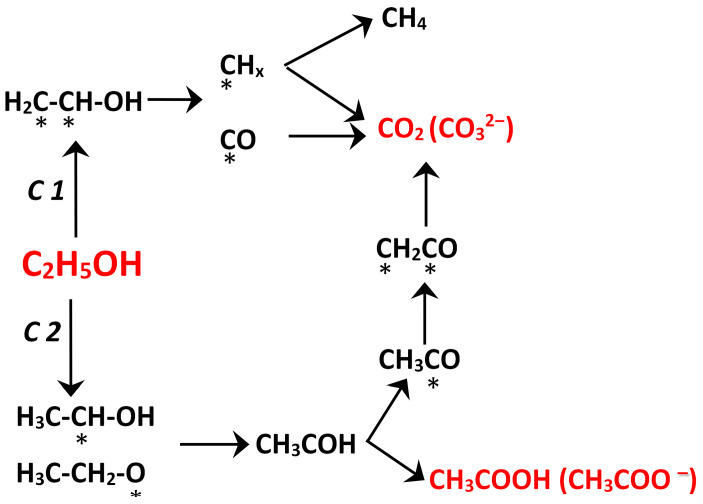
Pathways for the ethanol electrooxidation in the aqueous solutions and the main stable reaction products. Asterisks indicate the atoms via which the chemisorption proceeds.

The development of the catalysts for the electrooxidation of simple organic molecules occupies an important place in modern electrochemical materials science. A significant number of studies are devoted to the development of catalysts for the ethanol oxidation reaction (EOR). An active interest in this reaction is due to the prospects for using it in a wide range of electrochemical systems: sensors, fuel cells, and hydrogen generation electrolyzers [1]. As a current-generating reaction in a low-temperature direct ethanol fuel cell (FC), EOR is a very attractive process, because ethanol is one of the most energy-intensive and readily available fuels. Ethanol surpasses hydrogen and methanol in the volumetric energy density (21.7 MJ/L for liquid ethanol, 8 MJ/L for liquid hydrogen, and 16 MJ/L for liquid methanol). Therefore, the direct ethanol oxidation FCs are considered as promising electric generators for portable electronics and low- and medium-power vehicles. The development of such FC will make it possible to implement an environmentally friendly energy transformation cycle, because ethanol can be produced by the biosystems in unlimited quantities [2]. Recent works indicate that there remains a high scientific interest in the implementation of ethanol FC, despite the difficulties caused primarily by insufficient rate and depth of alcohol oxidation at low temperatures [1,3,4,5,6,7,8,9]. To achieve the theoretical characteristics of ethanol FC, it is necessary to provide the alcohol oxidation with breaking C–C bonds (Figure 1, pathway C1 [10,11,12,13,14]), whereas, in practice, EOR proceeds predominantly via the C2 pathway, yielding the incomplete oxidation products. It should be noted that the end product of EOR via the C2 pathway, acetic acid (or acetate ion), is not oxidized at low temperatures and can block the catalyst active sites [15]. Acetaldehyde can be oxidized to acetic acid or CO_2_, and the C–C bond energy in acetaldehyde is lower than in ethanol [12,16].

One of the key factors determining the selectivity and rate of ethanol oxidation is the nature of its chemisorption on the catalyst surface [2]. When a platinum electrode comes into contact with ethanol, the alcohol dehydrogenation and chemisorption occur. The chemisorption proceeds via the oxygen atom of the hydroxyl group and carbon atoms (Figure 1). Strong chemisorption of the alcohol molecule facilitates subsequent breaking of C–C bonds, and the process proceeds via the C1 pathway. The formation of weakly bound particles determines the preferential implementation of the C2 pathway.

For the effective EOR under the operating conditions of an anode of ethanol FC, the organic species adsorbed on the catalyst surface should be oxidized at the anodic potentials from 0.1 to 0.5 V (vs. reversible hydrogen electrode (RHE)). According to [13,17,18], the overall EOR rate in the potential range under investigation is limited by the chemical stages of interaction of the products of chemisorption and partial alcohol oxidation with oxygen-containing species:

CH_3_CO_ads_ + OH_ads_→ CH_3_COOH (1)

CO_ads_ + 2OH_ads_→ CO_2_ + H_2_O (2)

Due to a small number of active sites accessible for OH_ads_, the monoplatinum catalysts are characterized by a low EOR rate. The accumulation of the products of chemisorption and incomplete alcohol oxidation on the catalyst surface leads to its deactivation in the operating potential range of ethanol FC. One of the main approaches to enhance the efficiency of alcohol oxidation is the formation of multicomponent systems. The acceleration of EOR upon passing from the monometallic to multicomponent catalysts can be interpreted in terms of bifunctional catalysis [2,19,20]. The oxygen-containing species accumulate on the less noble metal or its oxide; these species are involved in the oxidation of organic species, which are chemisorbed on the platinum active sites. The principle of bifunctional catalysis is similarly realized in the case of CO adsorption and oxidation [21]. Thus, in the binary system, the properties of two components are used to provide a high catalyst activity and optimize their interaction with the reaction participants [3].

The following binary platinum-based catalysts for EOR are most studied: PtSn, PtRu, PtPd, and PtRh [20,22,23,24,25]. In most cases, an increase in the efficiency of EOR when passing from the monoplatinum catalysts to binary ones is associated with an increase in the process rate via the C2 pathway. A fraction of the process proceeding via the C1 pathway for the binary catalysts is frequently lower than for platinum [26]. A decrease in the alcohol oxidation depth may be due to a change in the nature of alcohol chemisorption, namely, the formation of predominantly weakly bound adsorbates in the case of the binary system [2]. When analyzing the regularities of EOR on the binary catalysts, the content of the non-noble component, its prevailing valence state, and the degree of alloying with platinum should be taken into account. In particular, the PtSn catalyst based on platinum metal and tin oxides is characterized by a higher CO_2_ yield as compared to the catalyst based on the PtSn alloy [22]. This is explained by the fact that platinum, with its unchanged crystal lattice parameter in the Pt–SnO_x_ system, ensures the formation of strongly bound species during alcohol chemisorption. The oxidation of these species is accompanied by the breaking of C–C bonds.

The electrolyte pH has a pronounced effect on the EOR [2,17,27]. When passing from the acidic solution to the alkaline solution, the rate of EOR increases. This is caused by a lower potential for the onset of formation of OH_ads_ oxygen-containing species. In addition, the presence of OH ions facilitates the detachment of protons from an alcohol molecule. It was shown that deeper dehydrogenation reduces the C–C bond dissociation energy [28]. The yield of the product of ethanol complete oxidation in the alkaline FC can reach 55% versus 2% in the case of a fuel cell with the proton-conducting (acidic) electrolyte [29]. From the viewpoint of EOR electrocatalysis, the corrosivity of the electrolyte is lower in the case of alkaline FC, allowing the use of the catalysts with reduced noble metal content.

The analysis of publications on the development of anodic catalysts for EOR shows continuing interest in creating platinum-based binary catalysts [30,31,32]. However, few studies have been devoted to the development of systems such as PtW and PtMo, although their effectiveness for this reaction was demonstrated [32,33,34,35,36]. These studies were typically conducted using the acidic electrolytes, and the acceleration of alcohol electrooxidation was observed when passing from monoplatinum to binary catalysts. In addition, it was indicated that molybdenum oxides played a positive role in dispersing platinum particles to form a catalyst with a high active surface area.

In this work, we synthesize and comprehensively examine the structural and electrochemical characteristics of Pt–Mo catalyst with varying platinum content. The study involves evaluating the catalyst activity toward EOR under the model conditions in the alkaline solution and testing the membrane–electrode assembly (MEA) of an alkaline ethanol fuel cell. The literature lacks studies on the effect of platinum content on the performance of the Pt–Mo catalyst in EOR, nor are there examples of testing this catalyst in the alkaline ethanol FC. However, such studies are crucial for optimizing a practically valuable catalyst. It should be noted that only one work was devoted to the study of this catalyst in EOR in an alkaline solution [36]. Unlike previously published works, carbon nanotubes (CNTs) are used as the catalyst support. From the viewpoint of EOR, the nanotubes offer advantages over the conventional supports (carbon blacks). They combine a high specific surface area with an increased content of meso- and macropores [37]. The structure of CNTs favors the transport of alcohol and its oxidation products within the anode active layer.

## 2. Materials and Methods

### 2.1. Methods of Catalyst Synthesis

The procedure of synthesis of the CNT-supported Pt–Mo anodic catalyst was described earlier [38]. Briefly, the precursors (ammonium molybdate and hexachloroplatinic acid) at a given ratio (a nominal atomic ratio of Pt:Mo = 1:1 was used) were dissolved in the water, and an aqueous CNT suspension and an excess amount of a reducing agent (formic acid) were added. The mixture was thoroughly mixed, evaporated on a water bath, and the dry residue was heat-treated at a temperature of 440 °C in an argon flow for 2 h. CNTs (Nanotechcenter LLC, Tambov, Russia; >99.0 wt %, S_BET_ > 270 m^2^/g) were pre-treated in an alkaline solution and used as the catalyst support [39,40]. For comparison, Pt/CNT monoplatinum catalyst was synthesized by a similar procedure.

For testing the ethanol–air FC, a catalyst based on the nitrogen-doped CNT (CNT_N_) was used as a component of the cathode active layer. To synthesize CNT_N_, the doping agent (melamine) was mixed with CNT, ground, and the resulting mixture was processed at 600 °C for an hour in an argon atmosphere according to the procedure described in [38].

### 2.2. Methods of Structural Studies

The morphology of the synthesized catalysts was studied using a Helios G4 Plasma FIB Uxe dual-beam scanning electron microscope (SEM) (FEI, Eindhoven, The Netherlands) with a through-lens detector and immersion optics. The energy-dispersive X-ray spectra were obtained using an EUMEX spectrometer (Heidenrod, Germany).

Possible alloys in the composition of Pt–Mo catalysts were detected using the X-ray diffraction analysis (XRD). The measurements were performed with an EMPYREAN diffractometer (Almelo, The Netherlands) in the Bragg–Brentano (reflection) geometry using CuKα radiation. The calculations took into account the wavelengths of 1.5406 and 1.5444 Ǻ with a 2:1 intensity ratio in the doublet. The diffraction patterns were treated, and the phases were identified using HighScore software (version 5.3a) and the International Centre for Diffraction Data (ICDD) database.

The valence state of the elements in the catalysts was studied by X-ray photoelectron spectroscopy (XPS) using an OMICRON ESCA+ spectrometer (Taunusstein, Germany) with an aluminum anode and an XM1000 monochromated AlKα X-ray source (a radiation energy of 1486.6 eV and a power of 252 W). The spectra were recorded using an Argus hemispherical detector–analyzer with an analyzer transmission energy of 50 eV for the overview spectrum and 20 eV for individual element spectra. The pressure in the analyzer chamber did not exceed 10-9 mbar. All spectra were accumulated at least three times. The fluctuation of the peak positions did not exceed ±0.3 eV. The spectra were separated into the components after subtracting the background, which was determined by the Shirley method [41]. The Scofield sensitivity coefficients were used for the quantitative analysis [42].

The platinum content in the synthesized catalysts was refined using an independent optical method with a SPECORD M40 spectrophotometer (Jena, Germany). The method is based on measuring the optical density of a solution of platinum complex with stannous chloride over a wide range of platinum concentrations [39].

### 2.3. Methods of Electrochemical Study

The electrochemical properties of the catalysts were studied in a three-electrode glass cell with a stationary disk glassy carbon electrode with a working surface area of 0.126 cm^2^. A water–alcohol suspension was prepared for the catalytic layer formation. It was applied onto the electrode at a rate of 150 μg (catalyst)/cm^2^. The measurements were performed using a mercury oxide reference electrode (all potentials in the text and figures are given against RHE) in the KOH solution with an addition of ethanol in various concentrations.

The Pt–Mo catalysts of various compositions were characterized by estimating the electrochemically accessible surface area (S_Pt_) in the 0.1 M KOH electrolyte deaerated with argon. S_Pt_ was determined by the cyclic voltammetry based on the hydrogen adsorption–desorption peaks, as described in [40]. The polarization curves of ethanol oxidation were measured on the stationary electrode with a scan rate of 5 mV/s from the open-circuit potential, which was reached after purging the solution with argon for 30 min.

To fabricate the active layers of the cathodes and anodes for the ethanol FC, polytetrafluoroethylene (PTFE) (VOMO, Shanghai, China) with a particle size of 0.5–1.0 μm was used as a binder. The amount of PTFE introduced was 20 wt % of the weight of carbon material in the catalyst. The suspension for forming the electrode active layers was prepared by the ultrasonic homogenization of a specified amount of the catalyst and PTFE in a 50:50 isopropanol/water mixture. After the ultrasonic treatment, the resulting homogeneous suspension was applied onto the microporous side of Sigracet 39 BB gas diffusion layer using the drop method. The geometric surface area of the electrodes was 5 cm^2^. The anodic catalysts were applied as calculated for a platinum loading of 1.0 mg_Pt_/cm^2^. Nitrogen-doped CNT (CNT_N_) [38] was used as the cathode electrocatalyst with a loading of 1.0 mg/cm^2^ in all cases. The prepared electrodes were dried for 15 min at 150 °C in a muffle furnace to remove residual solvent and improve adhesion between the catalyst and binder particles. After drying, the electrodes were placed into a 1 M KOH solution for 24 h. The MEA was tested in the ElectroChem cells (Woburn, MA, USA). During assembling the cells, the electrodes were separated by a hydrophilic PVDF separator 125 μm thick with pores up to 0.45 μm in diameter (Durapore HVLP04700, Merck Millipore, Darmstadt, Germany). The MEA was sealed using Teflon gaskets to a compression ratio of approximately 20%.

During testing of the MEA of the ethanol FC, 1 M KOH + 1 M C_2_H_5_OH fuel mixture was fed into the anodic compartment using a peristaltic pump at a rate of 50 mL/min. Oxygen was fed into the cathodic compartment at a rate of 100 mL/min with no excess pressure. The tests were performed at temperatures of 20–60 °C. The voltage and current were measured with a SmartStat PS-80 potentiostat (Elins, Chernogolovka, Russia).

## 3. Results and Discussion

### 3.1. Structural Studies

Catalysts with various platinum contents were studied, namely 20Pt/CNT, 10PtMo/CNT, 19.6PtMo/CNT, 27.2PtMo/CNT and 42.8PtMo/CNT. The catalyst designations indicate the mass content of platinum determined spectrophotometrically. Further, for simplicity, catalysts are designated as Pt (20Pt/CNT), PtMo-1 (10PtMo/CNT), PtMo-2 (19.6PtMo/CNT), PtMo-3 (27.2PtMo/CNT), PtMo-4 (42.8PtMo/CNT).

**Figure 2:**
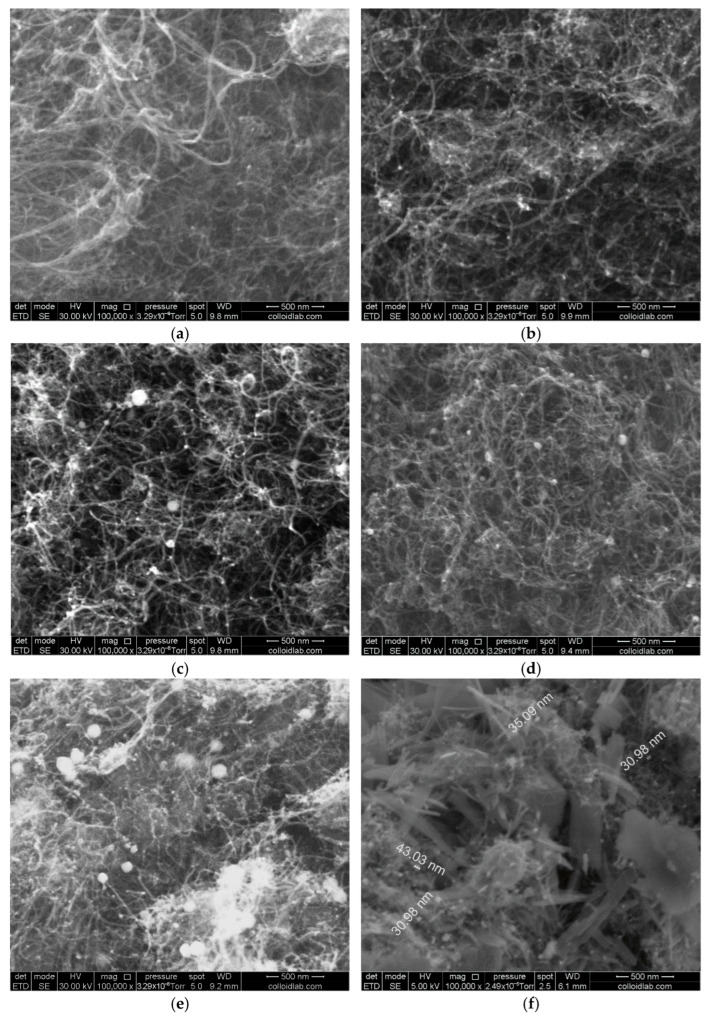
SEM images of test materials: (**a**) CNT; (**b**) Pt; (**c**) PtMo-1; (**d**) PtMo-2; (**e**) PtMo-3; (**f**) PtMo-4.

Figure 2 shows the SEM images of the test materials. Table 1 lists the contents of the elements on the specimen surface according to the data of energy-dispersive X-ray microanalysis (EDX).

The SEM images show a typical nanotube structure (Figure 2a), which is conserved during the catalyst formation. The crystalline structures are observed on the surface of the catalyst containing 42.8 wt % platinum; their presence may be due to incomplete washing of the catalyst. On the other hand, this specimen, which is characterized by a high oxygen content, contains the same elements as other PtMo systems (Table 1). This suggests that the observed crystals correspond to the molybdenum oxide compounds with individual clusters measuring 30–40 nm. It should be noted that the platinum contents, which were determined by X-ray microanalysis and spectrophotometry, agree well. The Pt:Mo atomic ratio corresponds to the 1:1 ratio specified based on the precursor weights for the PtMo-1 system. For systems with a higher platinum content, the atomic ratio Pt:Mo > 1:1. This may be due to a partial decoration of molybdenum oxide with platinum nanoparticles.

**Figure 3:**
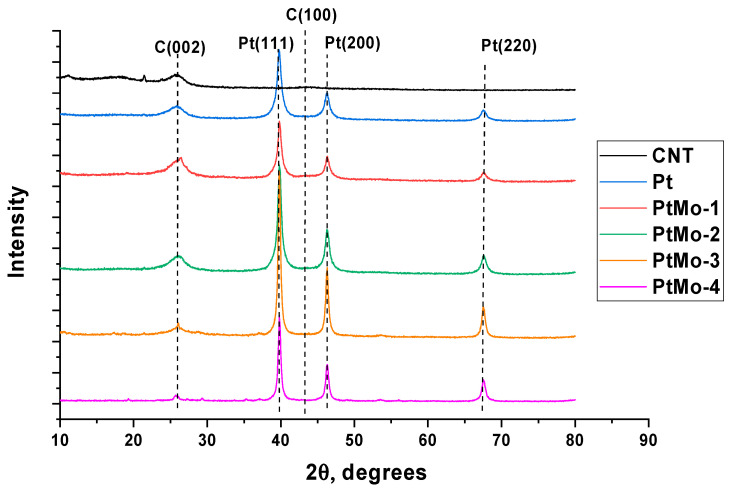
X-ray diffraction patterns of test materials.

Figure 3 shows the results of X-ray diffraction analysis of the catalysts. All diffraction patterns exhibit a characteristic peak of carbon C(002) at 2θ ≈ 26° corresponding to the interplanar spacing of graphitized carbon. This peak decreases with increasing platinum content in the catalyst. This is consistent with the SEM data that show shielding of the CNT surface by the crystalline structures in the case of the PtMo-4 catalyst. A weak peak corresponding to the C(100) face in the graphite lattice is observed only for the support material. The characteristic peaks of platinum at 2θ ≈ 39.8°, 46.4° and 67.6° [43] are observed both in the cases of monoplatinum catalyst and binary systems. The most intense peaks are observed for PtMo-2 and PtMo-4. It should be noted that the platinum peaks in the binary systems are almost not shifted along the 2θ axis relative to the corresponding peaks for the monoplatinum catalyst, indicating the absence of alloy formation. No individual molybdenum peaks are observed in the diffraction patterns. This may be due to the X-ray amorphous structure of its compounds and is consistent with the literature data [36].

**Figure 4:**
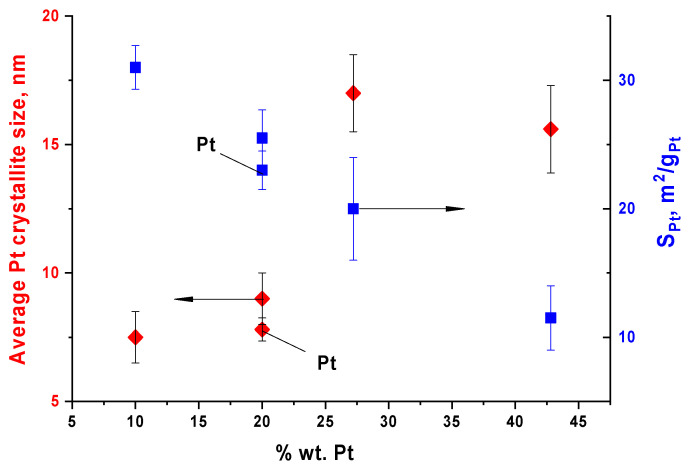
Dependences of the average platinum crystallite size and electrochemically accessible surface area on the platinum content in the PtMo/CNT system and Pt monoplatinum catalyst (indicated as Pt).

Figure 4 shows the dependence of the average platinum crystallite size (calculated from the Pt(220) half-peak width, as described in [44]) and S_Pt_ on the platinum content in various catalysts.

As can be seen, S_Pt_ decreases with increasing crystallite size. This is the evidence for a correlation between the crystallite and platinum nanoparticle sizes. The smallest crystallites, as expected, correspond to the catalyst with the lowest platinum content (PtMo-1), which is also characterized by the highest S_Pt_. It should be noted that the characteristics of the Pt and PtMo-2 catalysts are identical within the measurement errors. This indicates the absence of shielding of platinum by the molybdenum compounds. It should also be noted that measurement errors increase with increasing platinum content. This may be due to the increased heterogeneity of the samples, which is qualitatively confirmed by SEM images.

**Figure 5:**
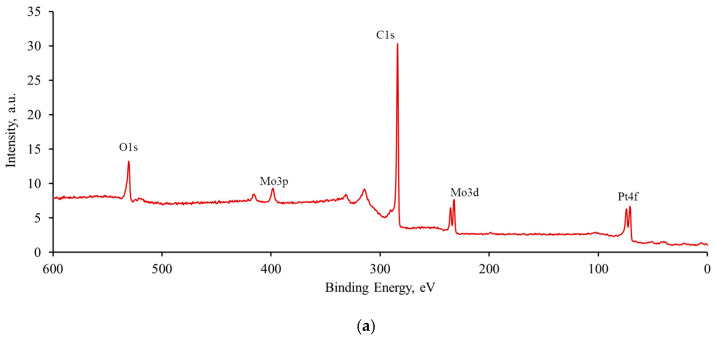

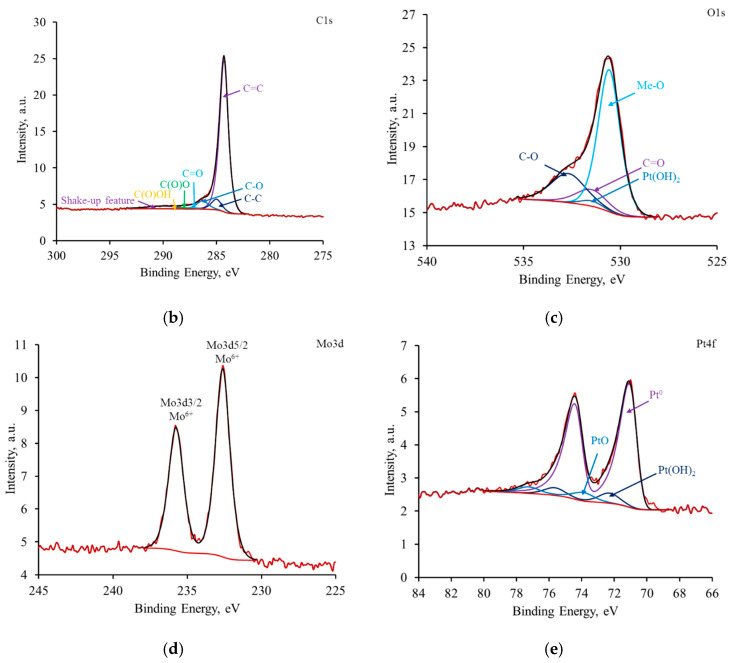
XPS spectra by the example of PtMo-4 catalyst: (**a**) overview spectrum, (**b**) C1s, (**c**) O1s, (**d**) Mo3d, and (**e**) Pt4f. The red line under peaks means the background line.

Figure 5 and Table 2 present the results of the XPS study of the catalyst surface composition. These data show that platinum is predominantly metallic on the surface of the studied materials, whereas molybdenum is hexavalent, probably in the form of MoO_3_ oxide. When forming PtMo/CNT catalysts, the electronic structure of platinum remains unchanged compared to Pt/CNT. This is evidenced by the constancy of the binding energy, which in both cases is 71.2 ± 0.1 eV for Pt(0)4f. This may contribute to the implementation of bifunctional catalysis in the oxidation of ethanol on the Pt-Mo catalyst rather than an electronic effect [45].

A higher platinum content relative to the molybdenum content may indicate partial decoration of molybdenum oxide with platinum particles, which is consistent with the EDX data. On the other hand, there is a discrepancy between the atomic ratios determined by XPS and EDX methods, which is especially evident in the case of heavy elements (Pt and Mo). This discrepancy may be partly due to the different depth of analysis, namely 1–10 nm for XPS and 1–5 µm for EDX. In addition, the XPS method is highly sensitive to impurities that can screen the signal from the target heavy element, reducing the intensity of its peaks. Therefore, XPS data should be considered as a source of information about the valence state of these elements rather than about their quantitative content in samples.

An independent study of material structure was performed using Raman spectroscopy. The corresponding results are presented in the Supplementary Materials.

### 3.2. Electrochemical Study

#### 3.2.1. Experiments Under the Model Conditions

In preliminary experiments, the stability of Pt-Mo catalysts (using the PtMo-2 system as an example) was assessed by the cyclic voltammetry method in KOH solutions in the absence of ethanol [39]. After cycling, the catalyst activity in the EOR was measured. It was shown that in 0.1 M KOH, the catalysts remain stable even when cycling over a wide potential range (0.1–1.3 V). In the range of potentials of the alcohol anode (0.1–0.5 V), the catalysts are stable even at a higher KOH concentration (0.5–1 M).

**Figure 6:**
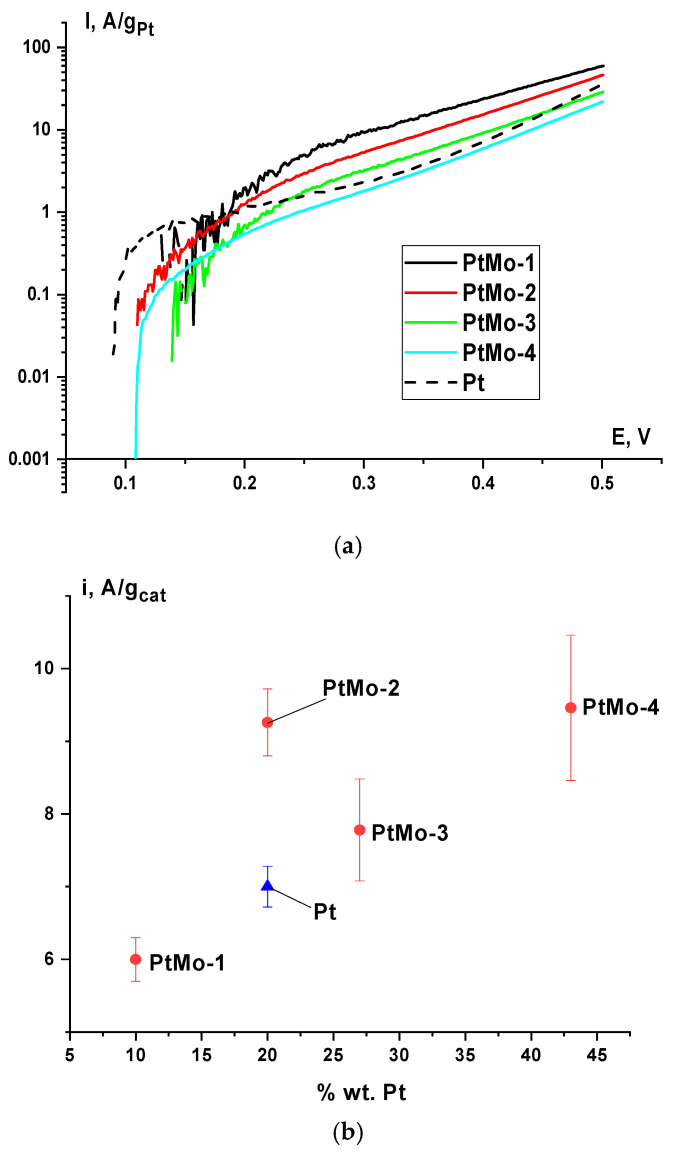
(**a**) Polarization curves of ethanol oxidation on PtMo/CNT catalysts (per platinum weight) with various platinum content and Pt. (**b**) Dependence of activity per catalyst weight at a potential of 0.5 V on the platinum content in PtMo/CNT and Pt catalysts; 0.1 M KOH +1 M C_2_H_5_OH solution.

Figure 6a shows the polarization curves characterizing the ethanol oxidation reaction rate (per platinum weight) on the test catalysts. As can be seen, the efficiency of platinum use for the EOR decreases with increasing platinum content in the catalyst. These data correlate with a decrease in the platinum surface area in the series 10PtMo > 19.6PtMo > 27.2PtMo > 42.8PtMo (Figure 4). Close slopes of the linear sections of the curves are evidence for a common mechanism of ethanol electrooxidation on the PtMo catalysts of various compositions. The EOR curve on the monoplatinum catalyst differs considerably from the curves for the binary systems. In the range of low polarizations, the EOR curve on Pt/CNT is characterized by an abrupt increase in the current; then it reaches a plateau at the potentials of 0.13–0.15 V. It can be supposed that a slower reaction on the monoplatinum catalyst is due to its poisoning by strongly bound organic adsorbates. In the case of binary systems, this undesirable effect is not observed, which indirectly indicates that the EOR proceeds on their surface according to the mechanism of bifunctional catalysis [2,19,20].

One of the key characteristics of a fuel cell catalyst is its activity per catalyst weight, because the ohmic and transport voltage losses increase with increasing amount of the catalyst in the electrode active layer. From this viewpoint, PtMo-2 and PtMo-4 are the most effective systems (Figure 6b). They were chosen for testing as a component of the MEA of the ethanol fuel cell.

**Figure 7:**
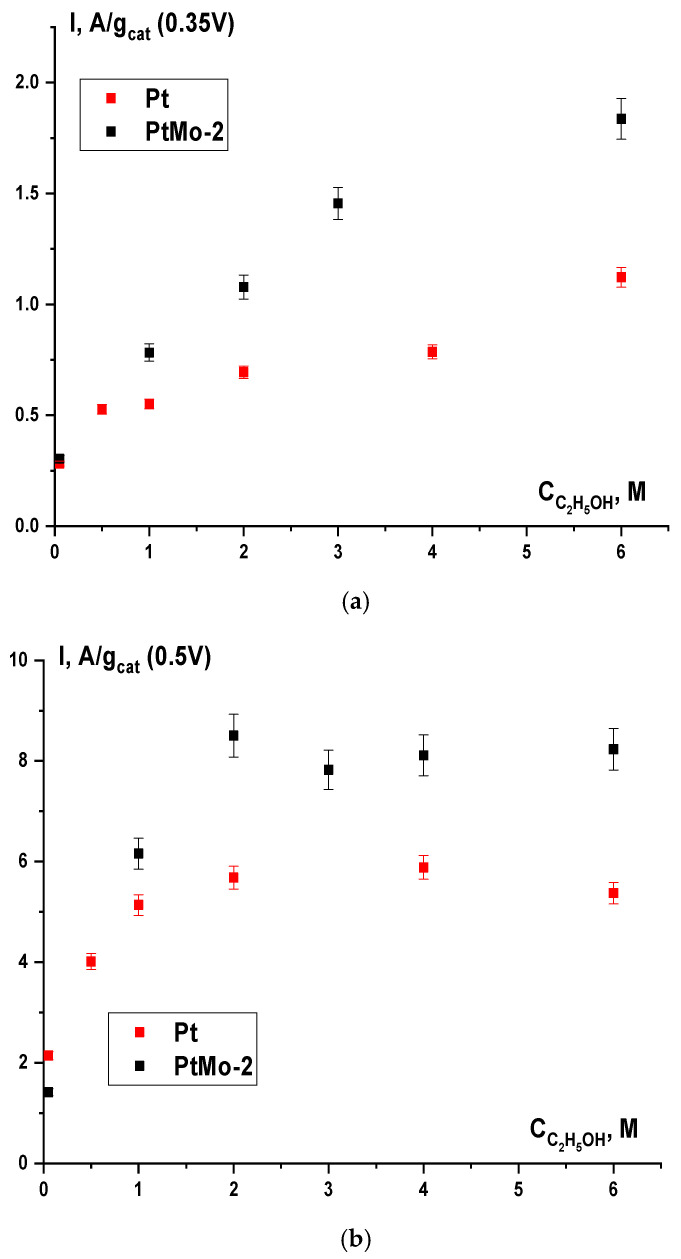
Dependences of the catalyst activity on the alcohol concentration in 0.1 M KOH solution at the potentials of (**a**) 0.35 V and (**b**) 0.5 V.

By the example of PtMo-2 and Pt catalysts, the dependence of the activity toward the EOR on the electrolyte composition was studied. As follows from the dependences shown in Figure 7, the catalyst activity at a potential of 0.35 V increases with increasing alcohol concentration in the 0.1 M KOH solution. At low ethanol concentrations (0.05–0.5 M), the catalysts exhibit similar activities, and the advantage of the binary catalyst over the monoplatinum one becomes evident at a concentration of 1 M and higher. With increasing polarization, the character of the studied dependences changes. At a potential of 0.5 V, for both catalysts, the dependences reach a plateau at an alcohol concentration of 2–6 M. Under these conditions, it is likely that the maximum filling of the platinum surface with the products of ethanol dissociative chemisorption is reached. In the case of the binary system, a higher oxidation current of these products is observed. This corresponds to the principle of bifunctional catalysis and is explained by a higher concentration of active oxygen-containing species on the surface compared to the monoplatinum catalyst.

**Figure 8:**
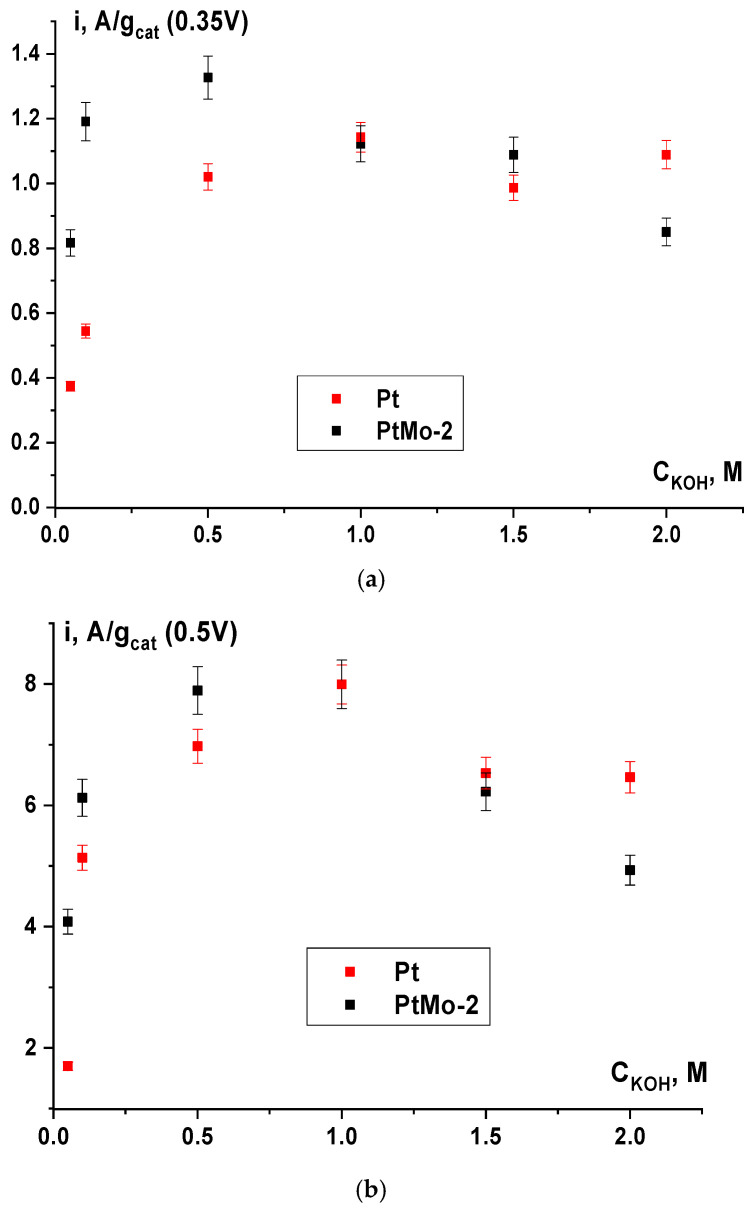
Dependences of the catalyst activity on the alkali concentration in 1 M C_2_H_5_OH solution at the potentials of (**a**) 0.35 V and (**b**) 0.5 V.

Figure 8 shows the dependence of activity on the alkali concentration in a 1 M ethanol solution for these catalysts. For both catalysts, the dependences pass through a maximum, which corresponds to an alkali concentration of 0.5 M, both at the potentials of 0.35 V and 0.5 V. At a higher alkali concentration, probably, partial blocking of the active sites accessible for ethanol chemisorption takes place. It should be noted that the PtMo-2 catalyst has an advantage over the monoplatinum catalyst only at an alkali concentration of up to 0.5 M. In the 1–2 M KOH solutions, the activity of the binary system decreases faster than in the case of Pt. It can be supposed that an excess amount of OH species is adsorbed on the surface of the PtMo-2 catalyst; this hampers the alcohol chemisorption even in 1 M KOH. In addition, the dissolution of molybdenum-containing components of the catalyst can take place in solutions with high alkali concentrations.

**Figure 9:**
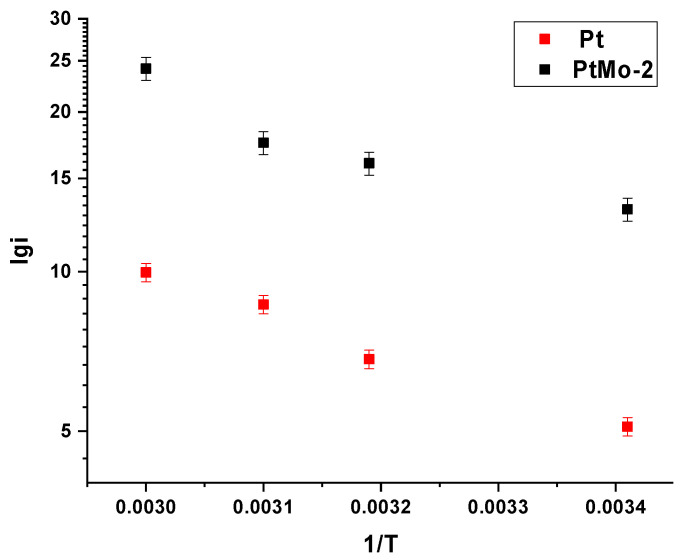
Dependences of current logarithm on 1/T at E = 0.5 V for the PtMo-2 and Pt catalysts; 0.1 M KOH + 1 M C_2_H_5_OH.

For the same catalysts, the temperature dependences of the EOR rate were measured over the operating temperature range of the alcohol FC (20–60 °C) (Figure 9). Based on these dependences, the EOR activation energies were calculated using the Arrhenius equation that relates the reaction rate constant to the activation energy:

(3) $$k=Ae^{-E_{a}/RT}$$

Assuming that the process mechanism remains unchanged with increasing temperature, the Arrhenius equation can be presented on the *lgi* = *f* (l/*T*) coordinates and the activation energy can be calculated:

(4) $$\frac{\partial lgi}{\partial (1/T)}=tg\;\alpha$$

(5) $$E_{a}=-2.303\times R\times \mathrm{tg}\;\alpha$$

For the current densities measured at a potential of 0.5 V, the activation energies *E*_a_ for PtMo and Pt are 11.4 and 13.8 kJ/mol, respectively. These results agree with *E*_a_ for platinum-containing catalysts reported in the literature [46] and are the evidence for the advantage of the binary catalyst over the monoplatinum one.

#### 3.2.2. Testing of MEA of Ethanol–Oxygen FC

**Figure 10:**
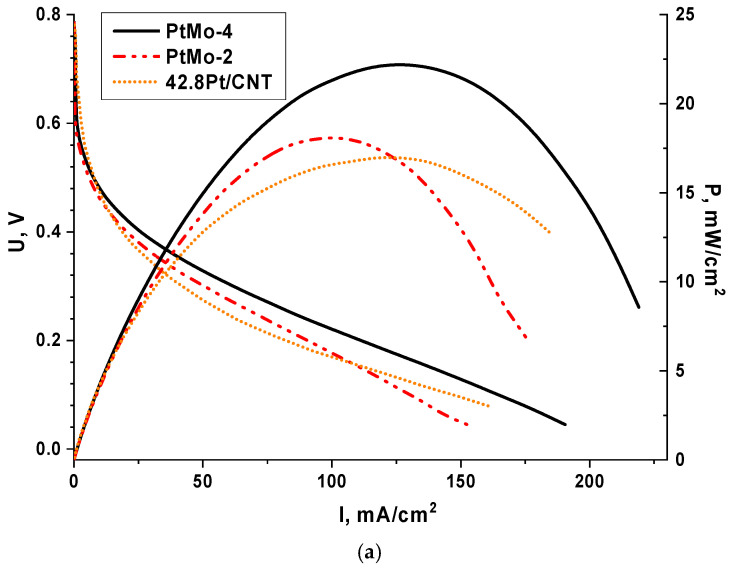

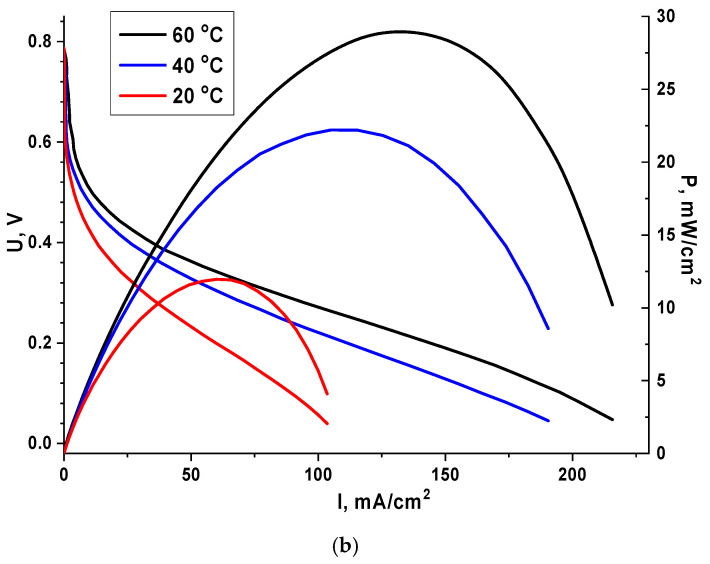
Voltammograms and dependences of power density on the current density for MEA of C_2_H_5_OH-O_2_ FC: (**a**) with various anodic catalysts at 40 °C, (**b**) with PtMo-4 anodic catalyst at various temperatures.

Figure 10 shows the results of testing the MEA of the ethanol-oxygen FC with various anodic catalysts. The advantage of the binary catalysts over the monoplatinum catalyst should be indicated. This is consistent with the results of the model experiments. When passing from the PtMo-2 catalyst to the PtMo-4 catalyst, an increase in the MEA characteristics is observed at the same platinum loading per unit geometric surface area of the anode (1 mg_Pt_/cm^2^). This effect can be explained by an increase in the number of platinum nanoparticles per unit volume of the active layer, because the carbon support loading was 4.1 and 1.34 mg_CNT_/cm^2^ for the PtMo-2 and PtMo-4 catalysts, respectively.

With increasing temperature, the characteristics of MEA increase reaching a maximum power density of 29 mW/cm^2^ at 60 °C (Figure 10b). It should be noted that this parameter was obtained with no excess oxygen pressure using a fuel mixture with relatively low concentrations of alcohol and alkali (1 M KOH + 1 M C_2_H_5_OH). In a number of works [47,48,49], the ethanol–oxygen FCs were tested under similar conditions and the maximum power densities ranged from 14 to 30 mW/cm^2^. Thus, the results obtained in this study correspond to the best parameters described in the literature.

## 4. Conclusions

The nanodispersed PtMo/CNT catalysts with various platinum contents are synthesized. Their structural characteristics and activity toward the ethanol electrooxidation reaction in the alkaline solution are studied. It is shown that over a wide range of platinum content (10–40 wt %), platinum in the catalyst is predominantly in the metallic state, whereas molybdenum is in the form of hexavalent oxide. The Pt:Mo atomic ratio increases (at a nominal ratio of 1:1) with increasing platinum content. This can be due to the decoration of molybdenum oxide with platinum nanoparticles. The kinetics of the ethanol electrooxidation reaction on the synthesized catalysts are studied under the model conditions. It is shown that the binary PtMo system outperforms the monoplatinum catalyst in the activity toward the EOR. The systems containing 20–40 wt % Pt exhibit the highest activity per catalyst weight. In testing of the ethanol FC with 42.8PtMo/CNT anodic catalyst, the maximum power density of 29 mW/cm^2^ was achieved, which corresponds to the level of the best parameters described in the literature under similar experimental conditions.

## Figures and Tables

**Table 1:** Concentration of elements on the surface of test materials according to the EDX data.

Material	Pt	Mo	C	O
	wt%//at.%	wt%//at.%	wt%//at.%	wt%//at.%
CNT	-	-	89.89//92.22	10.11//7.78
Pt	15.48//1.14	-	79.71//95.01	4.21//3.76
PtMo-1	10.85//0.81	5.20//0.79	74.19//89.57	9.75//8.84
PtMo-2	22.94//2.01	6.66//1.19	64.36//91.73	4.24//4.54
PtMo-3	22.10//1.84	4.20//0.71	67.19//90.84	6.51//6.61
PtMo-4	43.38//6.33	15.10//4.48	25.79//61.16	15.74//28.02

**Table 2:** Elemental composition of surface of test materials according to XPS data (at. %).

Material	O1s	C1s	Pt4f	Mo3d5/2
CNT	C-O—2.4% C=O—1.0%	C=C—91.2% C-C—2.6% C-O—1.9% C=O—0.4% C(O)O—0.3% C(O)OH—0.3%	-	-
Pt	C-O—2.3% C=O—1.2% Pt(OH)_2_—0.1%	C=C—89.3% C-O—3.6% C-C—1.4% C=O—0.7%	Pt^0^—0.6% Pt(OH)_2_—0.1%	-
PtMo-1	C-O—3.2% C=O—2.0% Me-O—0.9%	C=C—78.4% C-C—8.1% C-O—4.8% C=O—1.1%	Pt^0^—0.3%	Mo^6+^—0.2%
PtMo-2	C=O—2.4% C-O—1.6% Me-O—0.8%	C=C—87.0% C-C—3.0% C-O—1.9% C=O—1.8%	Pt^0^—0.6%	Mo^6+^—0.2%
PtMo-3	C-O—2.0% C=O—2.5% Pt(OH)_2_—0.1% Me-O—3.3%	C=C—84.8% C-C—1.3% C-O—2.1% C=O—1.6%	Pt^0^—0.6% Pt(OH)_2_—0.1% PtO—0.1%	Mo^6+^—0.7%
PtMo-4	C-O—3.3% C=O—1.7% Me-O—10.1% Pt(OH)_2_—0.4%	C=C—67.9% C-C—5.6% C-O—4.7% C=O—0.8% C(O)O—0.8%	Pt^0^—2.1% Pt(OH)_2_—0.2% PtO—0.2%	Mo^6+^—2.4%

## Data Availability

The original contributions presented in this study are included in the article/Supplementary Materials. Further inquiries can be directed to the corresponding author.

## Supplementary Materials

The following supporting information can be downloaded at: https://www.mdpi.com/article/10.3390/nano16090552/s1, Figure S1: Raman spectra for test materials; Table S1: Characteristics of test materials according to the Raman spectroscopy data. Ref. [50] is cited in the Supplementary Materials file.

## Abbreviations

The following abbreviations are used in this manuscript:

CNT	Carbon nanotube
EOR	Ethanol oxidation reaction
FC	Fuel cell
RHE	Reversible hydrogen electrode
SEM	Scanning electron microscope
XRD	X-ray diffraction analysis
ICDD	International Centre for Diffraction Data
XPS	X-ray photoelectron spectroscopy
CCD	Charged-coupled device
PTFE	Polytetrafluoroethylene
MEA	Membrane–electrode assembly
EDX	Energy-dispersive X-ray microanalysis
