# Supplementary material for: Effect of Platinum Content on Properties of CNT-Supported Pt–Mo Catalyst for Ethanol Electrooxidation Reaction
Source: Nanomaterials (Basel). 2026 Apr 30;16(9):552. doi: 10.3390/nano16090552 (PMC13164801; doi:10.3390/nano16090552)
Supplement: Supplementary file 1 [file nanomaterials-16-00552-s001.zip › nanomaterials-4281596-supplementary.pdf]

nanomaterials

MDPI

Supplementary Materials

Effect of Platinum Content on Properties of CNT-Supported Pt–Mo Catalyst for Ethanol Electrooxidation Reaction

Oleg Korchagin [1,2,*], Marina Radina [1], Alexey Kuzov [1], Vladimir Andreev [1,2] and Andzhela Bulanova [2]

1 Frumkin Institute of Physical Chemistry and Electrochemistry, Russian Academy of Sciences, Moscow 119071, Russia; merenkovamarina@mail.ru (M.R.); scourge@mail.ru (A.K.); vandr@phyche.ac.ru (V.A.)

2 Department of Physical Chemistry and Chromatography, Samara University, Samara 443086, Russia; av.bul@yandex.ru

* Correspondence: oleg-kor83@mail.ru

To evaluate possible changes in the support (CNT) structure as a result of the catalyst synthesis, the specimens were studied using the Raman spectroscopy. The Raman spectra were recorded in the backscatter mode using an InVia Renishaw spectrometer (ZEISS, Germany) equipped with a detector with a charged-coupled device (CCD), an ionic laser (λ = 532 nm), and a grating with a resolution of 1800 lines/mm with a spectral resolution of 1 cm^{-1}.

As shown in Figure S1, the spectra of all specimens exhibit three peaks characteristic of carbon materials: the D band of disordered carbon (~1350 cm^{-1}), the peak G of graphi-tized carbon (~ 1580 cm^{-1}) and 2D peak (~ 2690 cm^{-1}) [1]. The D band indicates the presence of structural defects and the ID/IG intensity ratio enables us to estimate a degree of mate-rial disorder. As is seen from Table S1, a degree of disorder increases upon passing from CNT to the catalysts, although no direct correlation is observed between the catalyst com-position and the I_D/I_G parameter. No noticeable shift of the D and G bands is observed during the catalyst formation. This indicates that the carbon support structure is retained, which is consistent with the SEM images.

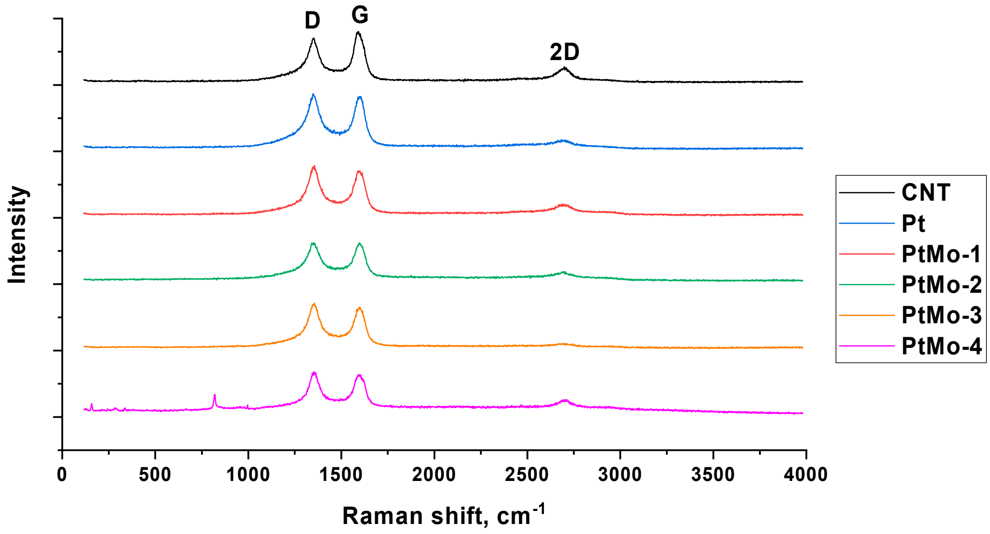

Figure S1. Raman spectra for test materials.

 https://doi.org/10.3390/nano16090552

Table S1. Characteristics of test materials according to the Raman spectroscopy data.

**Material**	**D, cm^{-1}**	**G, cm^{-1}**	**2D, cm^{-1}**	**I_D**	**I_G**	**I_{2D}**	**I_D/I_G**	**I_{2D}/I_G**
CNT	1353	1594	2698	1414	1603	549	0.88	0.340
Pt	1349	1599	2703	1735	1644	335	1.06	0.204
PtMo-1	1355	1601	2697	1580	1416	417	1.12	0.290
PtMo-2	1353	1597	2698	1246	1247	383	1.01	0.307
PtMo-3	1355	1600	2701	1437	1304	218	1.10	0.167
PtMo-4	1351	1599	2707	1359	1284	526	1.06	0.410

1. Lilloja, J.; Kibena-Põldsepp, E.; Sarapuu, A.; Kikas, A.; Kisand, V.; Käärik, M.; Merisalu, M.; Treshchalov, A.; Leis, J.; Sammelselg, V.; et al. Nitrogen-Doped Carbide-Derived Carbon/Carbon Nanotube Composites as Cathode Catalysts for Anion Exchange Membrane Fuel Cell Application. *Appl. Catal. B Environ.* **2020**, *272*, 119012, doi:10.1016/j.apcatb.2020.119012.
